# Glial Response and Neuronal Modulation Induced by Epidural Electrode Implant in the Pilocarpine Mouse Model of Epilepsy

**DOI:** 10.3390/biom14070834

**Published:** 2024-07-11

**Authors:** Giulia Spagnoli, Edoardo Parrella, Sara Ghazanfar Tehrani, Francesca Mengoni, Valentina Salari, Cristina Nistreanu, Ilaria Scambi, Andrea Sbarbati, Giuseppe Bertini, Paolo Francesco Fabene

**Affiliations:** 1 Section of Anatomy and Histology, Department of Neurosciences, Biomedicine, and Movement Science, School of Medicine, University of Verona, 37124 Verona, Italy; giulia.spagnoli@univr.it (G.S.); edoardo.parrella@univr.it (E.P.); sara.ghazanfartehrani@univr.it (S.G.T.); francesca.mengoni@univr.it (F.M.); cristina.nistreanu@studenti.univr.it (C.N.); ilaria.scambi@univr.it (I.S.); andrea.sbarbati@univr.it (A.S.); giuseppe.bertini@univr.it (G.B.); 2 Section of Innovation Biomedicine, Department of Engineering for Innovation Medicine, University of Verona, 37134 Verona, Italy; valentina.salari@univr.it

**Keywords:** epilepsy, pilocarpine, electrode implant, EEG, neuroinflammation, cytokines, microglia, astrocytes, glia

## Abstract

In animal models of epilepsy, cranial surgery is often required to implant electrodes for electroencephalography (EEG) recording. However, electrode implants can lead to the activation of glial cells and interfere with physiological neuronal activity. In this study, we evaluated the impact of epidural electrode implants in the pilocarpine mouse model of temporal lobe epilepsy. Brain neuroinflammation was assessed 1 and 3 weeks after surgery by cytokines quantification, immunohistochemistry, and western blotting. Moreover, we investigated the effect of pilocarpine, administered two weeks after surgery, on mice mortality rate. The reported results indicate that implanted mice suffer from neuroinflammation, characterized by an early release of pro-inflammatory cytokines, microglia activation, and subsequent astrogliosis, which persists after three weeks. Notably, mice subjected to electrode implants displayed a higher mortality rate following pilocarpine injection 2 weeks after the surgery. Moreover, the analysis of EEGs recorded from implanted mice revealed a high number of single spikes, indicating a possible increased susceptibility to seizures. In conclusion, epidural electrode implant in mice promotes neuroinflammation that could lower the seizure thresholds to pilocarpine and increase the death rate. An improved protocol considering the persistent neuroinflammation induced by electrode implants will address refinement and reduction, two of the 3Rs principles for the ethical use of animals in scientific research.

## 1. Introduction

The pilocarpine model represents one of the most widely used animal models of epilepsy [1]. Pilocarpine-treated rodents are models of both acutely induced seizures culminating with a prolonged episode of status epilepticus (SE) and of chronic spontaneous recurrent seizures (SRSs) that occur thereafter [2]. In rodents, the administration of pilocarpine, a muscarinic cholinergic agonist, induces ictal and interictal epileptic activity both in the hippocampus and neocortex, detectable by electrographic recordings. Such alterations are correlated with a sequence of behavioral signs, including akinesia, ataxic lurching, and facial automatisms, culminating with motor seizures and SE. The progression from pilocarpine administration to the development of SE, however, is not constant, as some animals never enter SE while others die following treatment. The outcome varies depending on species/strain and experimental protocols [2,3]. After several hours of SE (acute period), animals that survive remit spontaneously and proceed to a seizure-free period characterized by apparently normal behavior (latent period). This period, lasting up to 8 weeks, ends with the appearance of SRSs (chronic period). The frequency of SRSs during the chronic period also varies depending on the model, reaching up to several seizures per day [2,4].

Since the initial description of the model [5], various protocols have been proposed. Depending on the experimental aims, different laboratories use different animal species and strains, dosages, routes of administration, and pretreatment procedures [2]. In many experimental designs, pilocarpine treatment is preceded by cranial surgery to implant electrodes for acquiring electroencephalography (EEG) recordings [6,7,8,9,10,11]. EEG recordings allow the detection of the onset and progression of epilepsy by monitoring the frequency and duration of seizures, as well as the features of interictal activity [12].

Chronically implanted EEG electrodes can be ranked according to their invasiveness, from the least invasive, epidural, to subdural, intracortical, and finally depth electrodes [13]. However, even epidural electrode implants may trigger a tissue response that can interfere with the experimental outcomes [14]. Changes in the microenvironment surrounding the implant include mechanical stress and the “foreign body response”, an inflammatory reaction involving the activation of glial cells and the release of cytokines and chemokines [15,16,17]. In the long run, these responses may dramatically impact neuronal function, promote neuronal degeneration, and cause electrode failure [14,16].

Previous studies have investigated the effects of brain electrode implants in other epilepsy models, including rats subjected to electrical stimulation [18,19,20] and rodents treated with kainic acid (KA) [21,22]. On the other hand, the impact of surgically implanted cortical electrodes on the pilocarpine mouse model has never been studied. Here, we report the results of two experiments addressing: (1) the impact of epidural electrodes on the response of glial cells (i.e., microglia and astrocytes) in the underlying brain cortex, and (2) whether glial activation could affect mice condition following pilocarpine treatment.

## 2. Materials and Methods

### 2.1. Animals

Animal care and experimental procedures were conducted in accordance with the guidelines of the European Union directive 2010/63/EU. All protocols were approved by the local ethics committee (C.I.R.S.A.L, University of Verona) and the Italian Ministry of Health (protocol code 56DC9.60, authorization 570/2020-PR). Moreover, all the procedures were performed following the “Principles of Laboratory Animal Care” (NIH publication No. 86–23) to minimize the number of animals used and to avoid their suffering. NMRI male mice, acquired from Envigo (Udine, Italy), were housed in individually ventilated cages (IVCs), one animal per cage, with food and water ad libitum, and kept in a sound-attenuated room at constant temperature (22 ± 1.0 °C) and humidity (60 ± 5%) with a 12/12-h light-dark cycle with lights on at 7:00 a.m.

### 2.2. Experimental Design

In Experiment 1, epidural electrode implant surgeries were carried out (see below). After either 1 or 3 weeks of recovery, animals were sacrificed, and several biochemical and histological parameters of glial response were investigated in samples of brain tissue obtained from implanted vs. non-implanted animals. The responses were also compared to those evoked by the administration of lipopolysaccharide (LPS), a known proinflammatory agent.

In Experiment 2, after the same implant surgery and post-operative recovery, epilepsy was induced by systemic injection of pilocarpine. The impact of the chronic presence of electrodes on the severity of the condition was assessed by comparing survival in implanted vs. non-implanted mice.

In Experiment 3, implanted animals were treated with pilocarpine or vehicle, and their EEG activity was monitored.

A total of 83 mice, 8 weeks of age, were recruited for the study and randomly assigned to one of the following groups (Table 1):

Animals in the SUR group received the epidural electrode implant surgery described below, while SHAM mice were subjected to the same surgical procedure, except that electrodes were not implanted. Finally, the CTL group consisted of control animals who received no treatment but were sacrificed at the same time as their respective cohorts. The 2 LPS mice received a single systemic dose of LPS and were sacrificed 24 h later.

In Experiments 2 and 3, mice either received N-methylscopolamine bromide (NMS) followed by administration of pilocarpine (pilocarpine treatment) or NMS followed by administration of saline solution (vehicle treatment).

### 2.3. Epidural Electrode Implant Surgery

**Figure 1:**
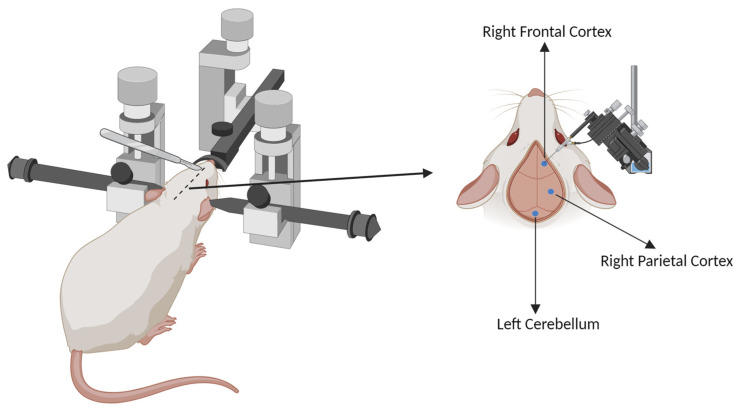
Diagram of electrode placement. The head of anesthetized mouse was immobilized in a stereotaxic frame, shaved, and the subcutaneous surface exposed. Three holes were gently drilled in the bone skull at the level of the right frontal cortex, right parietal cortex, and left cerebellum. Figure created with BioRender.com.

Animals from the SUR and SHAM groups were anesthetized with isoflurane (2% for induction and 1.3% during surgery) using an isoflurane vaporizer (Ugo Basile S.R.L, Varese, Italy). The anesthetized mice were placed on a stereotaxic frame (Stoelting Co., Wood Dale, IL, USA), the head was shaved with an animal trimmer, and a longitudinal incision was performed to expose the subcutaneous surface. By using a dental drill (Ideal Micro Drill, Fine Science Tools, Foster City, CA, USA), three holes (0.5 mm burr) were carefully drilled in the bone skull at the following coordinates, relative to bregma: 1.37 mm anterior and 1.23 mm lateral (i.e., over the right frontal cortex); 2.15 mm posterior and 1.98 mm lateral, (right parietal cortex); and 6.14 mm posterior and 0.85 mm lateral (left cerebellum) [23] (Figure 1).

In SUR (but not in SHAM) animals, 3 electrodes were inserted into the drilled holes. Electrodes consisted of a stainless-steel screw (1.6 mm, M1, 2 × 2.8 mm) with a silver wire soldered to the screw head and were manually positioned such that the tip of the screw touched the dura mater. The electrodes were then secured to the skull with self-curing methacrylate resin (Duracrol^®^, SpofaDental a.s., Markova, Czech Republic). Following surgery, the animals were treated subcutaneously (s.c.) with an analgesic (carprofen, Rimadyl^®^, 1 mL/kg, 1:10; Pfizer, Roma, Italy) and a wide-spectrum antibiotic (enrofloxacin, Baytril^®^, 10 mL/kg, 1:50, Bayer S.p.a., Milano, Italy) and were returned to their home cages. Analgesic and antibiotic administrations were repeated daily for the following two and three days, respectively.

### 2.4. LPS Administration

LPS (Sigma, Darmstadt, Germany) was injected intraperitoneally (i.p.) in a single 5 mg/kg dose, according to [24].

### 2.5. Brain Tissue Collection

Brain samples from all animals in Experiment 1 were examined post-mortem.

One-half of the mice in the SUR, SHAM, and CTRL groups (*n* = 18) were destined for immunohistochemical analyses. One or three weeks after surgery, these mice were deeply anesthetized by i.p. injection of 250 mg/kg 2,2,2-tribromoethanol (Merck KGaA, Darmstadt, Germany) and transcardially perfused with ice-cold 0.01 M phosphate-buffered saline (PBS) pH 7.4, followed by 4% paraformaldehyde (PFA, Boster, Pleasanton, CA, USA) in PBS. The brain was then removed, post-fixed in 4% PFA for 1 h, and stored for two days at 4 °C in a 30% sucrose solution in PBS for cryoprotection. Cryostat sections were cut at 20 μm and stored in PBS and sodium azide at 4 °C until processed for immunostaining.

The rest of the animals, including the LPS group (*n* = 20), were instead destined for biochemical assays. To obtain fresh brain samples, the mice were sacrificed by cervical dislocation, and the brains were immediately removed. The parietal cortex of both hemispheres was dissected, snap-frozen in liquid nitrogen, and stored at −80 °C until further processing.

### 2.6. Protein Extraction

Frozen parietal cortex samples were homogenized by tissue homogenizer in RIPA buffer “mild” (Tris-HCl 50 mM pH = 7.4, NaCl 150 mM, NP40 1%, Deoxycholic acid 0.5%) plus 1% protease inhibitor (Roche, Monza, Italy). All the samples were centrifuged at 14,000× *g* for 20 min, the supernatants were collected, and protein concentration was determined by the BCA assay (Thermo Fisher, Monza, Italy). The supernatant was then frozen and stored for subsequent cytokine measurements and western blot analysis.

### 2.7. Cytokine Measurement

An amount of 30 µg of protein lysates was used to quantify cytokines. Briefly, mouse LEGENDplex™ Multi-Analyte Flow Assay Kits (Biolegend, San Diego, CA, USA) are bead-based immunoassays used to identify concentrations of 13 inflammation-related cytokines/chemokines [IL-1α, IL-1β, IL-6, IL-10, IL-12p70, IL-23, IL-27, CCL2 (MCP-1), INF-β, IFN-γ, TNF-α, IL-4, and GM-CSF] in mice. The data were collected using a flow cytometer (LSRFortessa X-20, BD). The analysis was performed using LEGENDplex Data Analysis Software Version 8.0 (BioLegend). The results for protein lysates were normalized to their corresponding protein and presented as pg/mg of protein.

### 2.8. Western Blotting

Equal amounts of proteins (30 μg) for each sample were separated by SDS/PAGE in 4–20% gels (BioRad, Kabelsketal, Germany) and immunoblotted on Trans-Blot Turbo polyvinylidene fluoride (PVDF) transfer (BioRad, Kabelsketal, Germany). Membranes were incubated with primary antibodies in rabbit against glial fibrillary acidic protein (GFAP) (Dako, Glostrup, Denmark; 1:5000) and glyceraldehyde 3-phosphate dehydrogenase (GAPDH) (Thermo Fisher Scientific, Milan, Italy; 1:3000) overnight at 4 °C, then with HRP-conjugated secondary antibody (Invitrogen, Fisher Scientific, Milano Italy; 1:2000) for 2 h at room temperature. Finally, the membranes were incubated with a chemiluminescent reagent (ECL, WBLUR050, Luminata Crescendo Western HRP substrate, Merck KGaA, Darmstadt, Germany), and the signal was detected with the G:BOX F3 GeneSys system version 1.6.9.0 (Syngene, Bangalore, India). Data were analyzed with the ImageJ software version 1.54f using the gel analysis method. Densitometric analysis was performed using ImageJ software, normalizing GFAP bands to GAPDH levels as a control of equal loading of samples in the total protein extracts. Table S1 provides a list of the primary antibodies used in western blotting.

### 2.9. Immunohistochemistry

Bright-field immunohistochemistry was used to visualize cluster of differentiation 68 (CD68) and GFAP-positive cells in coronal brain sections. Sections were permeabilized and blocked in a solution of 0.3% Triton X-100 and 2% goat normal serum in PBS for one hour and then incubated in rat anti-CD68 (AbD SEROTEC, Kidlington, UK; 1:500) and rabbit anti-GFAP (Dako, Glostrup, Denmark; 1:500) overnight at 4 °C. The following day, samples were washed three times for 5 min each with 1× PBS and incubated with a biotinylated goat anti-rabbit IgG (GFAP) and goat anti-rat IgG (CD68), each diluted 1:200 for two hours at room temperature. Avidin–biotin–peroxidase solution (VECTASTAIN^®^ Elite ABC-HRP Kit, Vector Laboratories Inc., Burlingame, CA, USA) was used, and later the sections were stained with 3,3′diaminobenzidine (Merck KGaA, Darmstadt, Germany) and 0.75% hydrogen peroxide. Finally, slices were mounted with Entellan (Merck KGaA, Darmstadt, Germany). Representative images of CD68 and GFAP immunostaining in the parietal cortex were captured at the Olympus Bx51 using a Qimaging QIMAC camera by Image-Pro Plus 7.0 software. Table S1 provides a list of the primary antibodies used in immunohistochemistry.

### 2.10. Pilocarpine Treatment

Epilepsy was induced in all mice in Experiment 2 by pilocarpine administration. In the SUR group, the treatment took place two weeks after surgery. Mice received an i.p. injection of 1 mg/kg NMS (Sigma-Aldrich, Merck KGaA, Darmstadt, Germany), followed by an i.p. administration of 290 mg/kg pilocarpine hydrochloride (Sigma-Aldrich, Merck KGaA, Darmstadt, Germany) 30 min later. NMS was used to limit the peripheral cholinergic effects of pilocarpine [2]. The dosage of pilocarpine was based on a previous dose-response study with NMRI mice [25]. Behavioral observations were performed to assess SE during the first 6 h following pilocarpine administration. SE was defined as at least 30 min of continuous seizure activity scored between 3° and 5° Racine stages [26] with unresponsiveness to environmental stimuli and loss of postural control [25]. Survival was assessed until 12 days after pilocarpine treatment.

### 2.11. EEG Recording

EEG recording was performed in animals from Experiment 3, i.e., implanted mice treated with NMS followed by administration of saline solution (SUR + vehicle) and mice treated with NMS and pilocarpine (SUR + pilocarpine). EEG signals were acquired for 3 h starting right after the pilocarpine or vehicle injection and saved for offline analysis. The electrodes were connected to the g.BSamp amplifier (g.Tec medical engineering GmbH, Schiedlberg, Austria) and the 16-bit A/D convertor (Power8/35, AD Instruments, Ugo Basile S.R.L., Gemonio, Italy) by means of 6 ultra-flexible cables carrying EEG signals throughout a rotary interface (#AC6023, Moog, Elma, NY, USA), guaranteeing the animals’ maximum freedom of movement [9]. EEG recordings were digitally filtered (high-pass at 0.5 Hz, low-pass at 70 Hz, 50-Hz notch filter) and examined for the presence of “spikes” using the software LabChart v.8 (ADInstruments Pty Ltd., Bella Vista, Australia) [9]. EEG spikes were defined as high-voltage (>4 SD above background) positive or negative single deflections that lasted <50 ms. Each putative spike detected was confirmed by an expert observer by visual inspection.

### 2.12. Statistical Analysis

Statistical analysis was performed using the GraphPrism v.8 program (GraphPad Software Inc., San Diego, CA, USA). Comparison among groups was performed by two-way analysis of variance (ANOVA) followed by Sidak’s multiple comparison test. Survival upon pilocarpine treatment was analyzed by log-rank (Mantel–Cox) test. Confidence interval was fixed at 95%.

## 3. Results

### 3.1. Pro-Inflammatory Cytokines Are Increased in the Cortex of Mice Implanted with Electrodes 1 Week after Surgery

A panel of 13 cytokines was measured by LEGENDplex™ immunoassay in the parietal cortex (collected 1 or 3 weeks after surgery) of CTL, SHAM, and SUR mice. Three of the thirteen cytokines analyzed (i.e., IL-10, IL-17A, and GM-CSF) did not reach the detection threshold and could not be measured.

**Figure 2:**
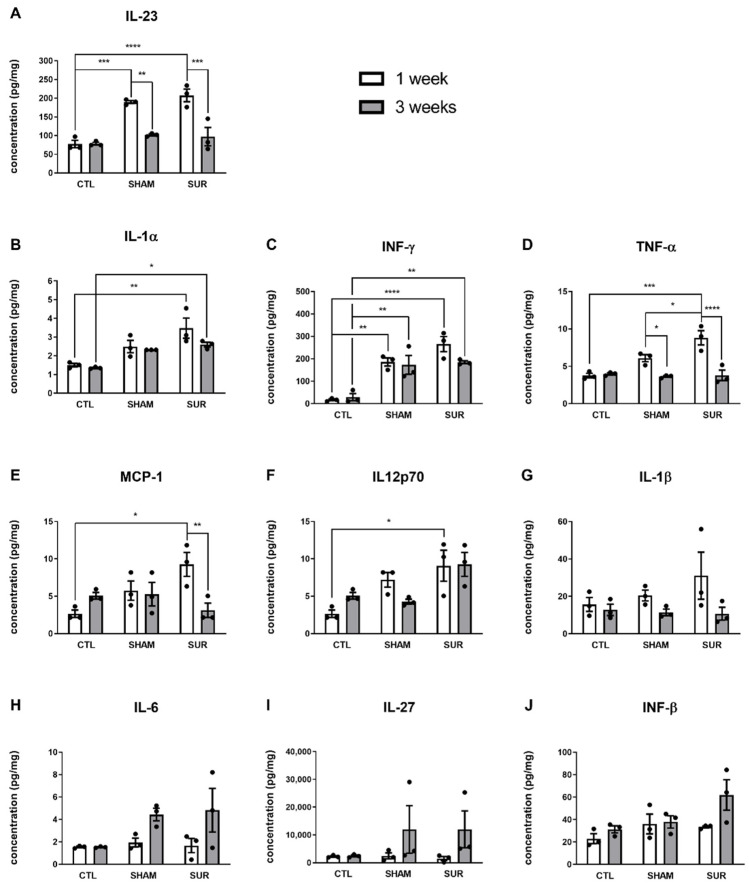
The effects of epidural electrode implant on cytokine release in parietal cortex. Cytokines were measured in the parietal cortex of CTL, SHAM, and SUR groups at 1 and 3 weeks after surgery. The concentrations of the following cytokines are shown: (**A**) IL-23), (**B**) IL-1α, (**C**) IFN-γ, (**D**) TNF-α, (**E**) MCP-1, (**F**) IL-12p70, (**G**) IL-1β, (**H**) IL-6, (**I**) IL-27, (**J**) IFN-β. *n* = 3 samples per group. * *p* < 0.05, ** *p* < 0.01, *** *p* < 0.001, **** *p* < 0.0001, 2-way ANOVA followed by Sidak’s multiple comparisons test.

One week after surgery, the levels of Il-23, IL-1α, IFN-γ, TNF- α, MCP-1, and IL12p70 in the SUR group were significantly higher when compared with the CTL group (CTL 1 week vs. SUR 1 week: *p* < 0.0001 Figure 2A; *p* < 0.01 Figure 2B; *p* < 0.0001 Figure 2C; *p* < 0.001 Figure 2D; *p* < 0.05 Figure 2E; *p* < 0.05 Figure 2F). Three weeks after surgery, the concentrations of IL-23, TNF-α, MCP-1, and IL12p70 came back to the levels of the CTL group (CTL 3 weeks vs. SUR 3 weeks: *p* > 0.05 Figure 2A,D–F; SUR 1 week vs. SUR 3 weeks: *p* < 0.001 Figure 2A; *p* < 0.0001 Figure 2D; *p* < 0.01 Figure 2E). Conversely, the concentrations of IL-1α and IFN-γ were still elevated after 3 weeks, although less markedly than at the 1-week time point (CTL 3 weeks vs. SUR 3 weeks: *p* < 0.05 Figure 2B; *p* < 0.01, Figure 2C).

**Figure 3:**
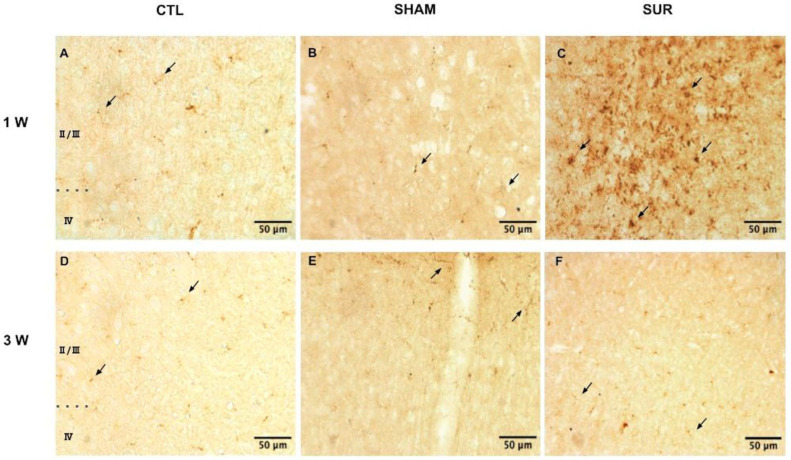
The effect of cortical electrode implant on CD68 immunoreactivity in the parietal cortex. Representative images of CD68 immunostaining in the parietal cortex of CTL (**A**,**D**), SHAM (**B**,**E**), and SUR (**C**,**F**) groups at 1 (**A**–**C**) and 3 weeks (**D**–**F**) after surgery. Note the higher level of CD68 immunoreactivity and activated phagocytic cells in the SUR group 1 week following electrode implantation (**C**). The cortical layers analyzed in this experiment are indicated in (**A**) and (**D**) (cortical layers II, III, and IV). Arrows indicate resting microglia in (**A**,**B**) and (**D**–**F**) and phagocytic cells in (**C**). Images are representative of three animals per group. Scale bar = 50 µm.

The surgical procedure without electrode implant promoted the increase of Il-23 and IFN-γ, although lightly with respect to electrode implantation. The effect was visible only 1 week after the surgery for IL-23 (CTL 1 week vs. SHAM 1 week, *p* < 0.001; Figure 2A) and 1 and 3 weeks after for IFN-γ (CTL 1 week vs. SHAM 1 week, *p* < 0.01; CTL 3 weeks vs. SHAM 3 weeks, *p* < 0.01; Figure 2C).

Finally, there were no significant effects of the surgery on the levels of IL-1β, IL-6, IL-27, and IFN-β (Figure 2G–J: CTL 1 week vs. SUR 1 week, *p* > 0.05; CTL 3 weeks vs. SUR 3 weeks, *p* > 0.05; CTL 1 week vs. SHAM 1 week, *p* > 0.05; CTL 3 weeks vs. SHAM 3 weeks, *p* > 0.05).

The statistical results described in Figure 2 are summarized in Table S2.

The LPS treatment was used as a positive control to assess the pro-inflammatory reaction in mice brains (Figure S1). As expected, LPS promoted a several-fold increase in the levels of different cytokines (Figure S1B,D,E,H,J), confirming the validity and reliability of the results obtained with this assay.

### 3.2. CD68 Immunoreactivity Increases in the Cortex of Mice Implanted with Electrodes 1 Week after Surgery

Neuroinflammation in the parietal cortex of CTL, SHAM, and SUR animals 1 and 3 weeks after surgery was also assessed by the qualitative analysis of CD68 immunostaining (Figure 3). CD68 is a lysosomal glycoprotein associated with the increased phagocytic activity of microglia and macrophages [27]. Few resting microglia with rod-shaped soma and long, thin ramified processes weakly positive for CD68 were detected in CTL and SHAM mice, as well as in SUR mice at 3 weeks (indicated by arrows in Figure 3A,B,D–F). CD68 immunoreactivity appeared augmented in the brains of SUR animals 1 week after the surgery. Here, some of the CD68-positive cells appeared to be large, round phagocytic cells that could be either activated microglia or blood-born macrophages (indicated by arrows in Figure 3C).

### 3.3. GFAP Levels Are Increased in the Cortex of Mice Implanted with Electrodes 3 Weeks after Surgery

**Figure 4:**
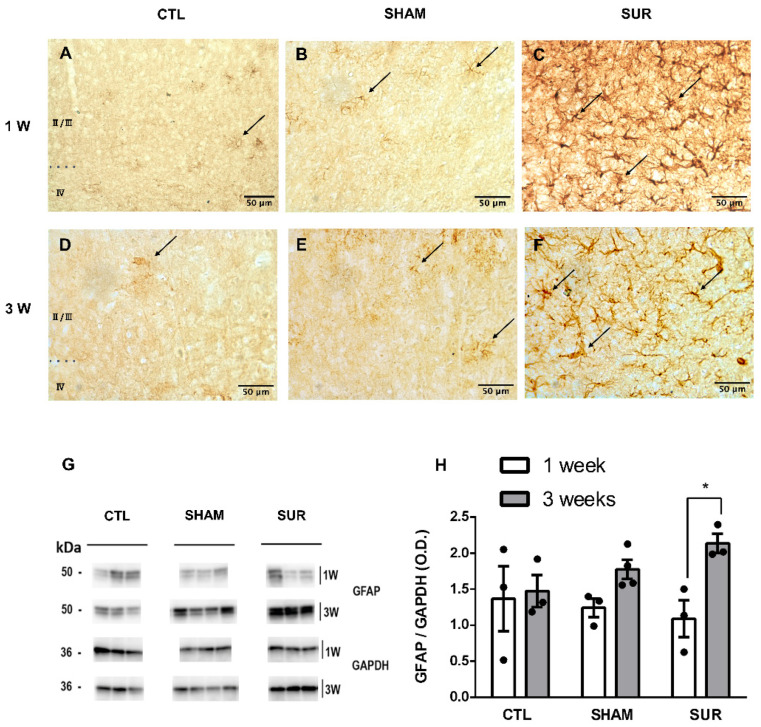
The effects of cortical electrode implant on GFAP levels in the parietal cortex. Representative pictures of GFAP immunostaining in the parietal cortex of CTL (**A**,**D**), SHAM (**B**,**E**), and SUR (**C**,**F**) groups at 1 (**A**–**C**) and 3 weeks (**D**–**F**) after surgery. Note the higher level of GFAP immunoreactivity in the SUR group at 1 and 3 weeks after electrode implantation. The cortical layers analyzed in this experiment are indicated in (**A**,**D**) (cortical layers II, III, and IV). Astrocytes are indicated by arrows. Images are representative of three animals per group. Scale bar = 50 µm. (**G**,**H**) Quantification of GFAP levels by western blot analysis. (**G**) Representative immunoblotting of GFAP and GAPDH in the cortex of CTL, SHAM, and SUR groups at 1 and 3 weeks after surgery. (**H**) Densitometry analysis showed an increase of GFAP levels in the SUR group at 3 weeks, although failing to reach statistical significance. GFAP expression in the SUR group was significantly higher at 3 weeks than 1 week after surgery (*n* = 3–4 samples per group. * *p* < 0.05, 2-way ANOVA followed by Sidak’s multiple comparisons test). Original Western blot images are available in Supplementary Materials.

Astrocytosis was assessed in the parietal cortex of CTL, SHAM, and SUR animals 1 and 3 weeks after the surgery by GFAP immunostaining and western blot. GFAP is the principal intermediate filament of astrocytes and is considered a biomarker of astroglial proliferation and astrogliosis [28]. GFAP immunostaining revealed an increase of immunoreactivity in the parietal cortex of mice subjected to electrode insertion 1 and 3 weeks earlier (Figure 4A–F). Indeed, the cortex of SUR animals presented several hypertrophic astrocytes with thick processes (Figure 4C,F), while the other experimental groups showed few cells with a stellate shape. Western blot analysis confirmed an increase in the GFAP levels of the SUR group compared with CTL 3 weeks after surgery only, although not significantly (Figure 4G,H: SUR 3 weeks vs. CTL 3 weeks, *p* > 0.05). Conversely, GFAP expression in the SUR group was significantly higher at 3 weeks than 1 week after the surgery (Figure 4G,H: SUR 3 weeks vs. SUR 1 week, *p* < 0.05). The statistical results described in Figure 4 are summarized in Table S2.

### 3.4. Mortality Rates after Pilocarpine Treatment Are Higher in Mice Implanted with Electrodes

**Figure 5:**
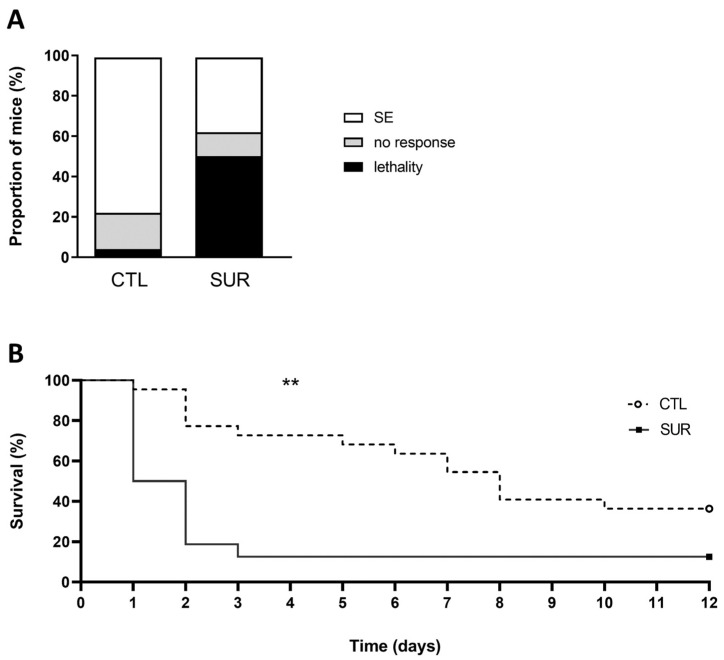
The effect of cortical electrode implant on pilocarpine action. (**A**) The graph represents the percentage of CTL and SUR mice displaying SE (white bars), no response (grey bars), and mortality (black bars) observed 6 h after administration of 290 mg/kg pilocarpine. (**B**) Survival of pilocarpine-injected CTL and SUR mice. A significantly higher number of implanted mice died the first days after treatment. ** *p* < 0.01, Log-rank (Mantel-Cox) test. CTL: *n* = 22 mice; SUR: *n* = 16 mice.

Next, we investigated whether electrode implants could impact the effect of pilocarpine. CTL and SUR mice were treated with pilocarpine (290 mg/kg) 2 weeks after surgery and monitored for the first 6 h following the drug injection. In accordance with previous studies [25], pilocarpine treatment on CTL mice provided a high incidence of SE (77%), with low percentages of animals showing mortality (5%) or no response (18%) (Figure 5A). Conversely, many implanted mice developed severe seizures and died in the first hours following pilocarpine administration (mortality rate 50%; Figure 5A). More SUR mice succumbed in the following 12 days compared to CNT mice (Figure 5B: survival rate CTL mice vs. SUR mice, *p* < 0.01). The statistical results described in Figure 5 are summarized in Table S2.

### 3.5. EEG Activity in Mice Implanted with Electrodes and Treated with Pilocarpine or Vehicle

**Figure 6:**
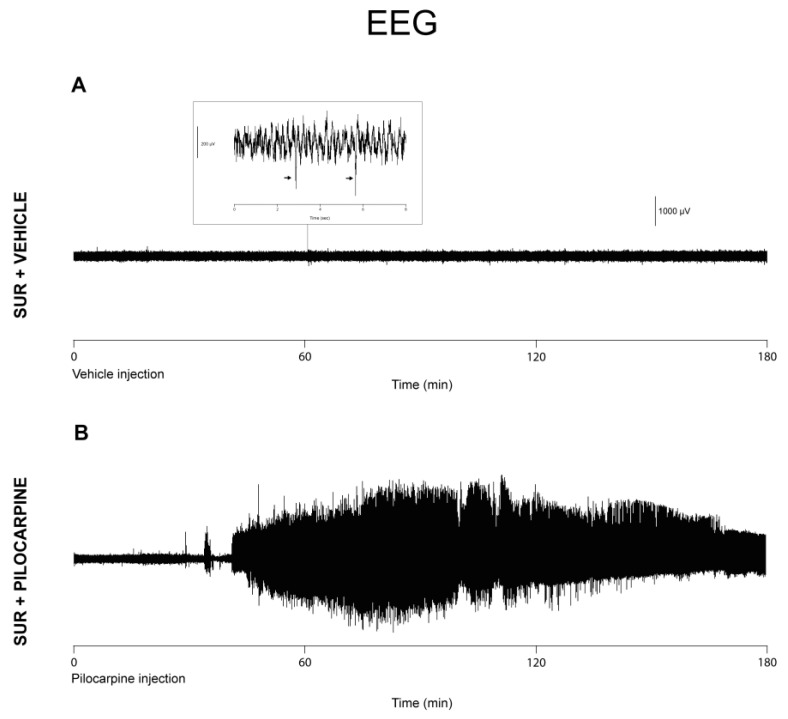
Representative samples of EEG activity recorded in implanted mice after administration of vehicle (SUR + vehicle, (**A**)) and pilocarpine (SUR + pilocarpine, (**B**)). Note the paroxysmal EEG activity following pilocarpine administration (**B**). Implanted mice that did not receive pilocarpine showed several single spikes of lower amplitude, two of which are shown in the expanded segment (**A**).

Finally, in order to investigate the causal relationship between neuroinflammation and seizure severity, we studied the EEG profile in implanted mice treated with vehicle and pilocarpine. SUR mice were treated with NMS followed by the administration of saline solution (SUR + vehicle) or pilocarpine 290 mg/kg (SUR + pilocarpine) 2 weeks after surgery, and their EEG activity was monitored for the first 3 h following the drug injection. In line with other pilocarpine studies [25], between 30 and 60 min after drug administration, some of the mice from the SUR + pilocarpine group displayed an electrographic activity characterized by high amplitude and high frequency paroxysmal discharges (Figure 6B). This EEG pattern corresponded with the sequence of continuous clonic-tonic seizures associated with loss of postural control consistent with SE (see Figure 5A). Conversely, mice from the SUR + vehicle group displayed several single spikes of low amplitude (Figure 6A).

## 4. Discussion

Although remarkably less invasive than other electrode surgeries, epidural implants are not free of side effects [29]. Studies on epidural implantation in rats and sheep showed thickening of the dura mater below the electrode and connective tissue overgrowth around it [30,31,32]. The implantation area was characterized by micro-hematomas, likely caused by irritation to the dural blood vessels during electrode insertion or by the pressure of the electrode itself on the soft and pulsating brain tissue [30]. In turn, the release of erythrocytes, platelets, and blood-serum proteins following the rupture of blood vessels can promote the recruitment of leukocytes and cause inflammation [33]. In support of this inflammatory scenario, astrocytic and microglial activation was observed in the superficial cortex layers of epidurally implanted animals [31,32].

Some of the findings presented in this research are essentially based on qualitative assessments, and we are aware that the low sample size of some experiments significantly limits the statistical power of the analyses. In spite of this caveat, in accordance with the studies reported above, the results clearly indicate that mice implanted with electrodes suffer from neuroinflammation sustained over time, consisting of an early pro-inflammatory cytokine release and microglia/macrophages activation, and a later astrogliosis.

The parietal cortex of mice subjected to electrode implantation displayed an increase of cytokines (namely Il-23, IL-1α, IFN-γ, TNF-α, MCP-1, and IL12p70) with pro-inflammatory properties [34,35,36,37,38]. Most of the studied cytokines were higher 1 week after surgery compared to 3 weeks after. Interestingly, other studies reported a similar increase of pro-inflammatory cytokines, including TNFα and IFN-γ, 1–2 weeks after the surgical implantation of intracerebral electrodes [22,39]. Of note, many cytokines induced by LPS treatment were several-fold higher than those measured following electrode insertion, indicating that implanted mice developed only mild neuroinflammation.

The peak of cytokine levels measured in the parietal cortex of SUR mice 1 week after surgery parallelled an increased immunoreactivity for CD68. CD68-positive cells could be either activated microglia or phagocytic cells derived from the bloodstream due to vascular damage [27]. Similarly, CD11b positive microglia/macrophages were detected in the cortex of sheep implanted with epidural electrodes [32].

As reported in previous studies [31,32], electrode insertion also promoted astrocytic activation, as showed by the increased GFAP levels measured in the parietal cortex by immunohistochemistry and western blot. Immunohistochemistry analysis indicated high levels of GFAP in the brain of mice implanted with the electrodes 1 and 3 weeks earlier, while western blot results suggested an increase of the protein only 3 weeks after the surgery. This apparent discrepancy in results can be caused by the different tissues analyzed with the two techniques. While immunohistochemistry displayed the astrogliosis in the cortex below the electrode, western blot may reflect the late astrocytic activation in cortical areas more distant from the insertion site.

The surgery-mediated neuroinflammation progression highlighted in this research is in accordance with the inflammatory reactions observed in other studies exploring the effect of intracranial electrode implants [14]. It has been shown that microglia in the proximity of the electrode become activated immediately after the surgery [40]. In the following hours and days, other microglia migrate toward the electrode and release pro-inflammatory molecules [15,16,40]. Conversely, astrocytes present a slower reaction than microglia, displaying a strong reaction only after several days. Interestingly, it has been demonstrated that the increase in GFAP levels is mainly caused by an upregulation of GFAP expression rather than a migration of astrocytes to the injury area [41].

Our results show that mice subjected to electrode implants are more sensitive to pilocarpine effects. Mice that underwent implantation developed more severe seizures than control animals and succumbed at a higher rate in the first hours and days following pilocarpine injection. An indication that the higher mortality in implanted mice was caused by a potentiated epileptogenic effect of pilocarpine comes from the study of EEG activity. Indeed, the analysis of EEGs recorded from mice implanted 2 weeks earlier revealed a high number of single spikes, potentially suggesting increased susceptibility to seizures. Of note, in other rodent models of epilepsy, cranial surgery procedures reduce the seizure threshold to epileptogenic stimuli, including electrical stimulation and KA treatment [18,19,20,21]. Over the past years, an increasing body of studies linked inflammation mechanisms, including the release of cytokines and vascular alterations, with the pathogenesis of epilepsy [42,43,44]. Here, we speculate that the lower seizure threshold to pilocarpine observed in the mice subjected to surgery could be caused by localized and persistent neuroinflammation resulting from the electrode implants.

In support of this, several studies have shown microglial over-activation with the concomitant production of pro-inflammatory molecules in the brains of epilepsy animal models and patients [45,46]. Pro-inflammatory cytokines are well-known to lower the seizure threshold [47,48]. In particular, IFN-γ and TNF-α, two pro-inflammatory cytokines that we found to be elevated in the parietal cortex of mice subjected to surgery, have been linked to epilepsy [49,50,51,52]. Also, the possible vascular damage caused by electrode implantation could play a role in the strengthening of the pilocarpine effect. Indeed, the exposure of neurons and astrocytes to blood-derived albumin or potassium ions has been shown to promote epileptiform activity [42]. Finally, reactive astrogliosis has been observed in epileptic foci [53]. Activated astrocytes undergo morphological, biochemical, and functional changes, including the disruption of ion and neurotransmitter homeostasis and alteration in intercellular communications, which can promote or exacerbate neuronal hyperexcitability [54,55,56].

## 5. Conclusions

In the pilocarpine mouse model of temporal lobe epilepsy, the mild but persistent neuroinflammation caused by epidural electrode implant may have a role in lowering the seizure threshold to pilocarpine. Therefore, in order to avoid bias in the EEG studies, to minimize the number of experimental animals, and to limit their suffering, it becomes crucial to take into account such inflammatory temporal distribution. An experimental design considering a longer recovery time after surgery or a more appropriate anti-inflammatory therapy could help to limit any interfering effect caused by the epidural electrode implant.

## Figures and Tables

**Table 1:** Experimental design. *: same mice.

	Experimental Groups				Timeline	Treatment
	SUR	SHAM	CTL	LPS		
Experiment 1	*n* = 6	*n* = 6	*n* = 6		surgery to sacrifice: 1 week	
	*n* = 6	*n* = 7	*n* = 6		surgery to sacrifice: 3 weeks	
				*n* = 2	LPS to sacrifice: 24 h	
Experiment 2	*n* = 16 *		*n* = 22		surgery to treatment: 2 weeks	pilocarpine
Experiment 3	*n* = 6				surgery to treatment: 2 weeks	vehicle
	*n* = 16 *				surgery to treatment: 2 weeks	pilocarpine

## Data Availability

The data are available from the corresponding author upon reasonable request.

## Supplementary Materials

The following supporting information can be downloaded at: https://www.mdpi.com/article/10.3390/biom14070834/s1, Figure S1: the effect of LPS injection on cytokine release in parietal cortex; Table S1: List of primary antibodies used for immunohistochemistry (IHC) and western blot (WB) analysis; Table S2: The statistical results of Figure 2, Figure 4, and Figure 5.
