# Supplementary material for: Glial Response and Neuronal Modulation Induced by Epidural Electrode Implant in the Pilocarpine Mouse Model of Epilepsy
Source: Biomolecules. 2024 Jul 11;14(7):834. doi: 10.3390/biom14070834 (PMC11274793; doi:10.3390/biom14070834)
Supplement: Supplementary file 1 [file biomolecules-14-00834-s001.zip › biomolecules-3016241-original-images.pdf]

Glial response and neuronal modulation induced by epidural electrode implant in the pilocarpine mouse model of epilepsy

Giulia Spagnoli[1†], Edoardo Parrella[2, 1†], Sara Ghazanfar Tehrani[1], Francesca Mengoni[1], Valentina Salari[2], Cristina Nistreanu[1], Ilaria Scambi[1], Andrea Sbarbati[1], Giuseppe Bertini[1,] and Paolo Francesco Fabene[2, 1*]

1 Section of Anatomy and Histology, Department of Neurosciences, Biomedicine, and Movement Science, School of Medicine, University of Verona, Verona, Italy.
2 Section of Innovation Biomedicine, Department of Engineering for Innovation Medicine, University of Verona, Verona, Italy.
† These authors contributed equally to this work
* Correspondence: paolo.fabene@univr.it

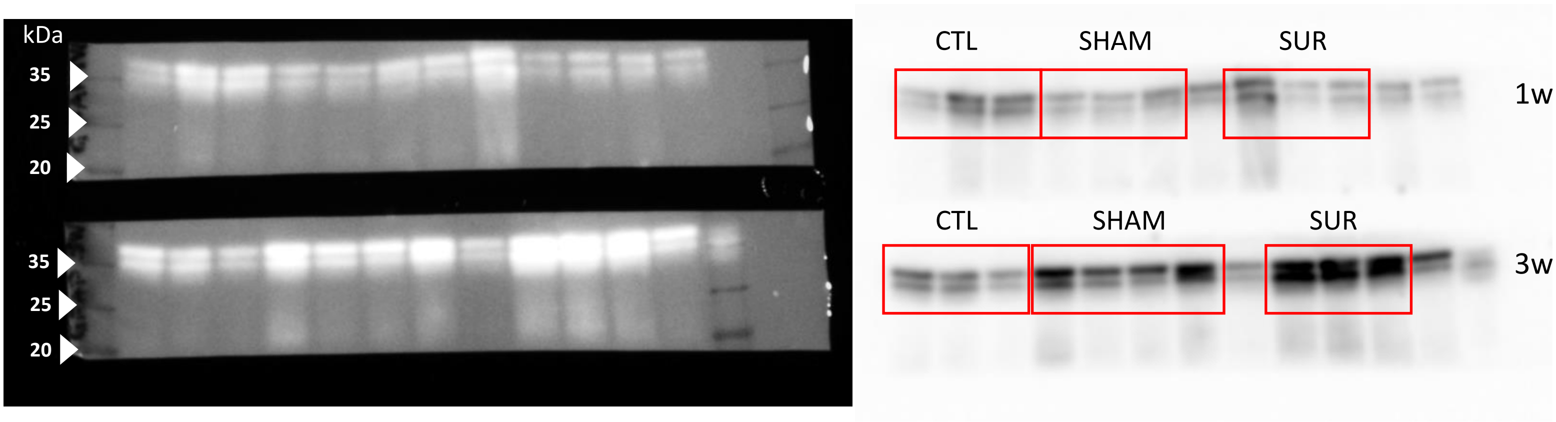

Blot 1: Western blot of GFAP from parietal cortex of CTL, SHAM and SUR groups. GFAP membrane are shown merged with the protein molecular weight marker (kDa) on the left. On the right are shown the full length western blots of gels presented in Figure 3h of the main article. The red line boxes referred to the cropped parts that are showed in the main article.

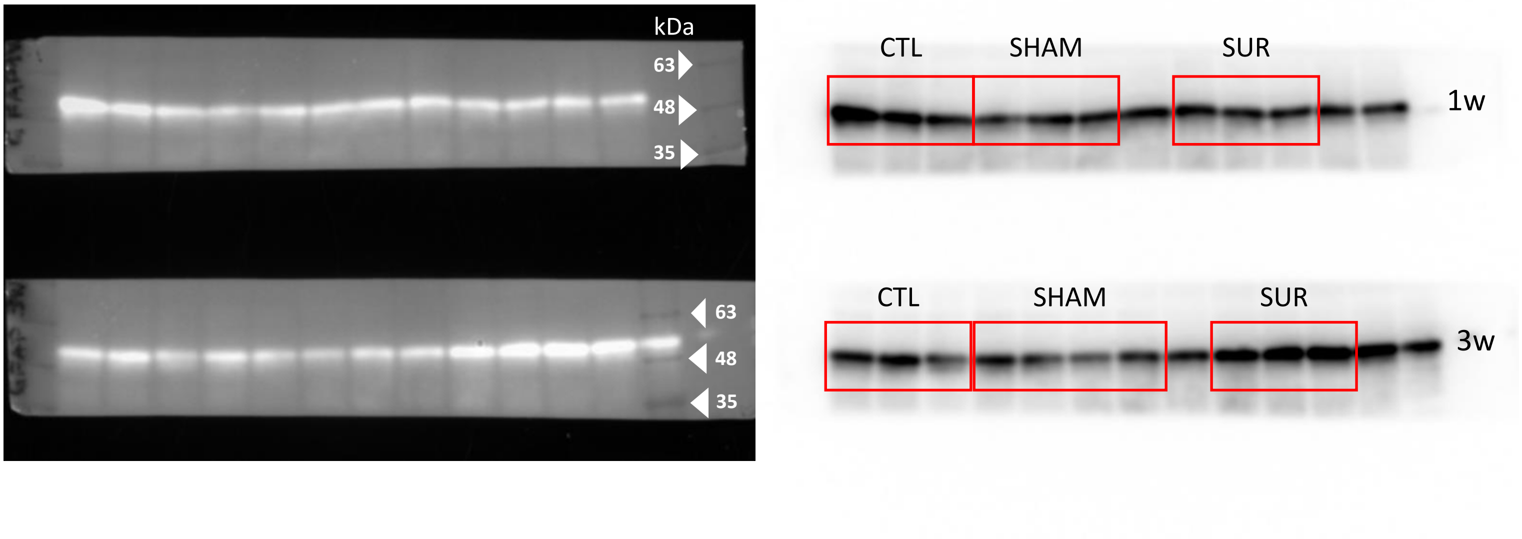

Blot 2: Western blot of GAPDH from parietal cortex of CTL, SHAM and SUR groups. GAPDH membrane are shown merged with the protein molecular weight marker (kDa) on the left. On the right are shown the full length western blots of gels presented in Figure 3h of the main article. The red line boxes referred to the cropped parts that are showed in the main article.
