# Supplementary material for: Glial Response and Neuronal Modulation Induced by Epidural Electrode Implant in the Pilocarpine Mouse Model of Epilepsy
Source: Biomolecules. 2024 Jul 11;14(7):834. doi: 10.3390/biom14070834 (PMC11274793; doi:10.3390/biom14070834)
Supplement: Supplementary file 1 [file biomolecules-14-00834-s001.zip › Supplementary Files/Table S1.pdf]

Table S1

List of primary antibodies used for immunohistochemistry (IHC) and western blot (WB) analysis.

**Primary antibodies, manufacturer**	**Catalogue number**	**Dilution**	**Secondary antibody**
anti-GFAP in rabbit, Dako	Z0334	1:500 (IHC)<br>1:5000 (WB)	Goat anti- rabbit
anti-CD68 in rat, AbD SEROTEC	MCA1957	1:500 (IHC)	Goat anti-rat
anti-GAPDH in mouse, Thermo Fisher Scientific	AM4300	1:3000 (WB)	Goat anti-mouse
