# Supplementary material for: Glial Response and Neuronal Modulation Induced by Epidural Electrode Implant in the Pilocarpine Mouse Model of Epilepsy
Source: Biomolecules. 2024 Jul 11;14(7):834. doi: 10.3390/biom14070834 (PMC11274793; doi:10.3390/biom14070834)
Supplement: Supplementary file 1 [file biomolecules-14-00834-s001.zip › Supplementary Files/Table S2.pdf]

Table S2
The statistical results of Figure 2, Figure 4 and Figure 5.
n.s.: not significant.

Figure 2

Figure 2A, IL-23 measurement.
Two-way ANOVA followed by Sidak's multiple comparisons test.

**1 week**	**significance**
CTL *vs.* SHAM	***
CTL *vs.* SUR	****
SHAM *vs.* SUR	n.s.

**3 weeks**	**significance**
CTL *vs.* SHAM	n.s.
CTL *vs.* SUR	n.s.
SHAM *vs.* SUR	n.s.

**1 week *vs.* 3 weeks**	**significance**
CTL	n.s.
SHAM	**
SUR	***

Figure 2B, IL-1α measurement.
Two-way ANOVA followed by Sidak's multiple comparisons test

**1 week**	**significance**
CTL *vs.* SHAM	n.s.
CTL *vs.* SUR	**
SHAM *vs.* SUR	n.s.

**3 weeks**	**significance**
CTL *vs.* SHAM	n.s.
CTL *vs.* SUR	*
SHAM *vs.* SUR	n.s.

**1 week *vs.* 3 weeks**	**significance**
CTL	n.s.
SHAM	n.s.
SUR	n.s.

Figure 2C, INF-γ measurement.
Two-way ANOVA followed by Sidak's multiple comparisons test

**1 week**	**significance**
CTL *vs.* SHAM	**
CTL *vs.* SUR	****
SHAM *vs.* SUR	n.s.

**3 weeks**	**significance**
CTL *vs.* SHAM	**
CTL *vs.* SUR	**
SHAM *vs.* SUR	n.s.

**1 week *vs.* 3 weeks**	**significance**
CTL	n.s.
SHAM	n.s.
SUR	n.s.

Figure 2D, TNF-α measurement.
Two-way ANOVA followed by Sidak's multiple comparisons test

**1 week**	**significance**
CTL *vs.* SHAM	n.s.
CTL *vs.* SUR	***
SHAM *vs.* SUR	*

**3 weeks**	**significance**
CTL *vs.* SHAM	n.s.
CTL *vs.* SUR	n.s.
SHAM *vs.* SUR	n.s.

**1 week *vs.* 3 weeks**	**significance**
CTL	n.s.
SHAM	*
SUR	****

Figure 2E, MCP-1 measurement.
Two-way ANOVA followed by Sidak's multiple comparisons test

**1 week**	**significance**
CTL *vs.* SHAM	n.s.
CTL *vs.* SUR	*
SHAM *vs.* SUR	n.s.

**3 weeks**	**significance**
CTL *vs.* SHAM	n.s.
CTL *vs.* SUR	n.s.
SHAM *vs.* SUR	n.s.

**1 week *vs.* 3 weeks**	**significance**
CTL	n.s.
SHAM	n.s.
SUR	**

Figure 2F, IL12p70 measurement.
Two-way ANOVA followed by Sidak's multiple comparisons test

**1 week**	**significance**
CTL *vs.* SHAM	n.s.
CTL *vs.* SUR	*
SHAM *vs.* SUR	n.s.

**3 weeks**	**significance**
CTL *vs.* SHAM	n.s.
CTL *vs.* SUR	n.s.
SHAM *vs.* SUR	n.s.

**1 week *vs.* 3 weeks**	**significance**
CTL	n.s.
SHAM	n.s.
SUR	n.s.

Figure 2G, IL-1β measurement.
Two-way ANOVA followed by Sidak's multiple comparisons test

**1 week**	**significance**
CTL *vs.* SHAM	n.s.
CTL *vs.* SUR	n.s.
SHAM *vs.* SUR	n.s.

**3 weeks**	**significance**
CTL *vs.* SHAM	n.s.
CTL *vs.* SUR	n.s.
SHAM *vs.* SUR	n.s.

**1 week *vs.* 3 weeks**	**significance**
CTL	n.s.
SHAM	n.s.
SUR	n.s.

Figure 2H, IL-6 measurement.
Two-way ANOVA followed by Sidak's multiple comparisons test

**1 week**	**significance**
CTL *vs.* SHAM	n.s.
CTL *vs.* SUR	n.s.
SHAM *vs.* SUR	n.s.

**3 weeks**	**significance**
CTL *vs.* SHAM	n.s.
CTL *vs.* SUR	n.s.
SHAM *vs.* SUR	n.s.

**1 week *vs.* 3 weeks**	**significance**
CTL	n.s.
SHAM	n.s.
SUR	n.s.

Figure 2I, IL-27 measurement.
Two-way ANOVA followed by Sidak's multiple comparisons test

**1 week**	**significance**
CTL *vs.* SHAM	n.s.
CTL *vs.* SUR	n.s.
SHAM *vs.* SUR	n.s.

**3 weeks**	**significance**
CTL *vs.* SHAM	n.s.
CTL *vs.* SUR	n.s.
SHAM *vs.* SUR	n.s.

**1 week *vs.* 3 weeks**	**significance**
CTL	n.s.
SHAM	n.s.
SUR	n.s.

Figure 2J, INF-β measurement.
Two-way ANOVA followed by Sidak's multiple comparisons test

**1 week**	**significance**
CTL *vs.* SHAM	n.s.
CTL *vs.* SUR	n.s.
SHAM *vs.* SUR	n.s.

**3 weeks**	**significance**
CTL *vs.* SHAM	n.s.
CTL *vs.* SUR	n.s.
SHAM *vs.* SUR	n.s.

**1 week *vs.* 3 weeks**	**significance**
CTL	n.s.
SHAM	n.s.
SUR	n.s.

Figure 4

Figure 4I, quantification of GFAP levels by western blot analysis.
Two-way ANOVA followed by Sidak's multiple comparisons test

**1 week**	**significance**
CTL *vs.* SHAM	n.s.
CTL *vs.* SUR	n.s.
SHAM *vs.* SUR	n.s.

**3 weeks**	**significance**
CTL *vs.* SHAM	n.s.
CTL *vs.* SUR	n.s.
SHAM *vs.* SUR	n.s.

**1 week *vs.* 3 weeks**	**significance**
CTL	n.s.
SHAM	n.s.
SUR	*

Figure 5

Figure 5B, survival of pilocarpine-injected CTL and SUR mice.
Log-rank (Mantel-Cox) test.

	**significance**
CTL *vs.* SUR	**
